# Supplementary material for: Physico-Chemical Characteristics of Spodumene Concentrate and Its Thermal Transformations
Source: Materials (Basel). 2021 Dec 3;14(23):7423. doi: 10.3390/ma14237423 (PMC8658623; doi:10.3390/ma14237423)
Supplement: Supplementary file 1 [file materials-14-07423-s001.zip › materials-1429248-supplementary.pdf]

SUPPLEMENTARY MATERIAL

Physico-chemical Characteristics of Spodumene Concentrate and its Thermal Transformations

Allen Yushark Fosu[1], Ndue Kanari [1], Danièle Bartier[1], Harrison Hodge[2], James Vaughan[2], Alexandre Chagnes[1,*]

[1] Université de Lorraine, CNRS, GeoRessources, F-54000 Nancy, France allen.fosu@univ-lorraine.fr (A.Y.F.); ndue.kanari@univ-lorraine.fr (N.K.); daniele.bartier@univ-lorraine.fr (D.B) ; alexandre.chagnes@univ-lorraine.fr (A.C.)

[2] School of Chemical Engineering, The University of Queensland, Brisbane, QLD 4072, Australia; james.vaughan@uq.edu.au

* Correspondence: alexandre.chagnes@univ-lorraine.fr; Tel.: +33(0)-372-744-544

Table S1: Calculated elemental assay of major elements by MLA and their corresponding oxides.

Element	Wt%	Oxides	Wt%
Al	11.58	Al2O3	21.87
Ca	1.94	CaO	2.76
Fe	2	Fe2O3	2.86
K	1.34	K2O	3.23
Li	1.96	Li2O	4.22
Mg	0.5	MgO	0.83
Mn	0.22	MnO	0.41
Na	0.76	Na2O	2.05
P	0.17	P2O5	0.22
S	0.07	SO3	0.18
Si	29.47	SiO2	63.15

Table S2: MLA data for degree of mineral liberation in concentrate used to generate Figure 4

Mineral	Liberation degree
Pyrite	98.3
Quartz	98.8
Orthoclase	93.7
Albite	98.2
Anorthite	97.2
Biotite	98.4
Muscovite	96.0
Chlorite	97.2
Amphibole	96.9
Spessartine	94.1
Spodumene	98.8
Tantalite_Mn	96.6
Calcite	95.8
Apatite	96.2

Table S3. Standard deviation on atomic percent of elemental composition of spodumene in concentrate.

**Element**	**Atomic %**													**SD***
Al	11.3	11.3	11.6	11.5	11.5	11.5	11.4	11.3	11.3	11.4	11.4	11.2	11.3	0.1036
Si	23.9	23.9	23.7	23.7	23.8	23.6	23.7	23.8	23.9	23.6	23.6	23.8	23.7	0.1029
O	64.8	64.8	64.7	64.7	64.7	64.7	64.7	64.8	64.8	64.7	64.7	64.8	64.7	0.0231
Fe						0.2		0.2		0.2	0.3	0.2	0.5	0.0539
Mn							0.2							

Table S4. Standard deviation on atomic percent of elemental composition of amphiboles in concentrate.

**Element**	**Atomic %**															**SD***
Mg	6.1	5.5	4.6	7.3	10.5	6.4	10.3	9.6	7.1	4.2	8.6	6.0	4.3		5.6	2.1346
Al	0.2	0.2	3.6	0.4	4.6	2.8	3.2	1.7	3.3	4.8	9.1		5.2	9.5		2.9863
Si	19.7	19.7	21.8	19.9	18.3	19.2	3.8	19.6	18.0	17.1	12.8	19.7	16.8	14.8	19.6	4.3916
Ca	9.6	9.6	3.2	9.5	0.4	5.0	0.3	4.7	5.0	3.9		9.6	4.8	0.2	9.7	3.6591
Fe	3.6	3.9	3.0	2.2	2.9	4.5	20.3	2.8	4.8	6.9	7.4	3.7	6.2	6.3	4.2	4.3969
O	60.8	60.9	62.1	60.6	60.4	61.2	58.4	60.8	60.7	61.0	60.1	60.8	60.8	61.3	60.9	0.7813
Na			0.8		1.3	0.5		0.4	0.8	0.9	1.0		1.1			0.2947
K			1.0		1.4	0.4		0.3	0.4	1.0	1.0		0.5			0.4113
Cr					0.3		2.5	0.2			0.2					1.1491
Mn						0.2	1.2			0.2		0.2	0.2	8.0		3.1233

Table S5. Standard deviation on atomic percent of elemental composition of quartz in concentrate.

Element	Atomic %									SD*
Si	33.3	33.3	33.3	33.3	33.3	33.3	33.3	33.3	33.3	33.3	0.0
O	66.7	66.7	66.7	66.7	66.7	66.7	66.7	66.7	66.7	66.7	0.0

Table S6. Standard deviation on atomic percent of elemental composition of albite in concentrate.

Element	Atomic %								SD*
Na	5.5	7.3	7.0	6.7	7.0	7.0	6.7	5.5	0.6861
Al	5.4	7.8	7.7	7.8	7.7	7.6	7.7	5.4	1.0706
Si	26.0	23.1	23.4	23.2	23.5	23.5	23.3	26.0	1.2354
O	63.0	61.7	61.9	61.8	61.9	61.9	61.9	63.0	0.5219
Ca	0.1	0.2	0.1	0.3			0.2	0.1	0.0579

Table S7. Standard deviation on atomic percent of elemental composition of mica in concentrate.

Element	Atomic %			SD*
Al	15.0	15.0	15.1	0.0557
Si	17.7	17.5	17.5	0.13
K	4.9	5.1	5.3	0.1823
Fe	0.9	0.9	0.8	0.0702
O	61.6	61.4	61.4	0.0985

Table S8. Standard deviation on atomic percent of elemental composition of hematite in concentrate.

Element	Atomic %					SD*
Fe	37.9	40.0	38.1	38.7	38.9	0.8418
O	60.1	60.0	60.2	60.2	60.1	0.0728
Si	1.3		1.0	1.0	0.6	0.2971
Ca	0.4					
Mn	0.4					
Al			0.8	0.4	0.4	0.2136

Table S9. Standard deviation on atomic percent of elemental composition of apatite in concentrate.

Element	Atomic %				SD*
F	7.9	8.1	7.9	8.6	0.3320
P	14.5	14.6	14.5	14.6	0.0455
Ca	20.4	19.8	20.1	20.2	0.2443
Mn	0.3	0.6	0.5		0.15875
O	56.9	56.9	57.0	56.6	0.1656

SD* represents the standard deviation on measured atomic percent of elements in mineral phases of the concentrate.

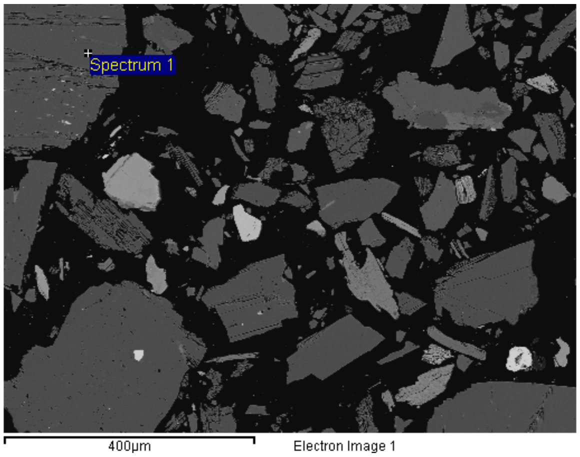

(a)

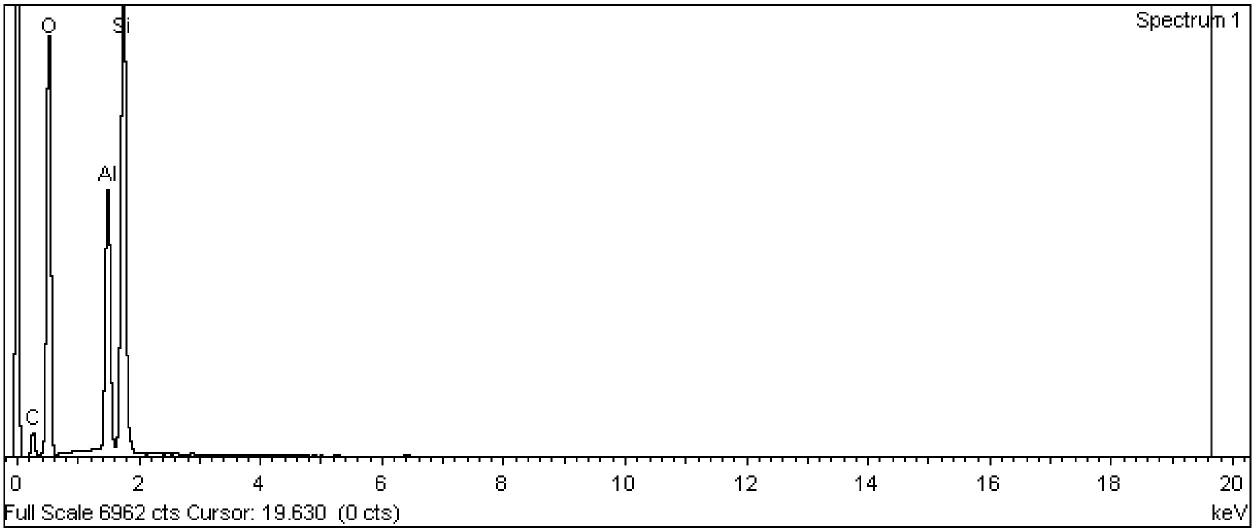

(b)

Figure S1: Spot analysis (a) and spectrum (b) of elemental composition of spodumene grain in concentrate.

Table S10: Raw data of spodumene grain generated by SEM-EDS for spectrum 1.

Element	App Conc.	Intensity Corrn.	Weight%	Weight% Sigma	Atomic%	Compd%	Formula	Number of ions
Al K	15.23	1.0706	14.23	0.14	11.32	26.88	Al2O3	1.40
Si K	29.53	0.9441	31.28	0.22	23.90	66.92	SiO2	2.95
O			48.29	0.27	64.78			8.00
Total			93.79					
							Cation sum	4.35

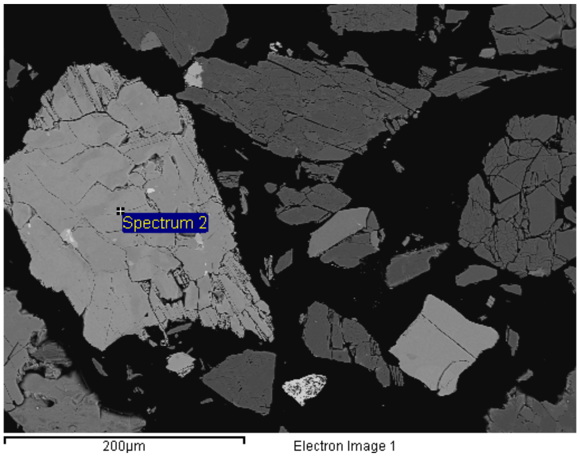

(a)

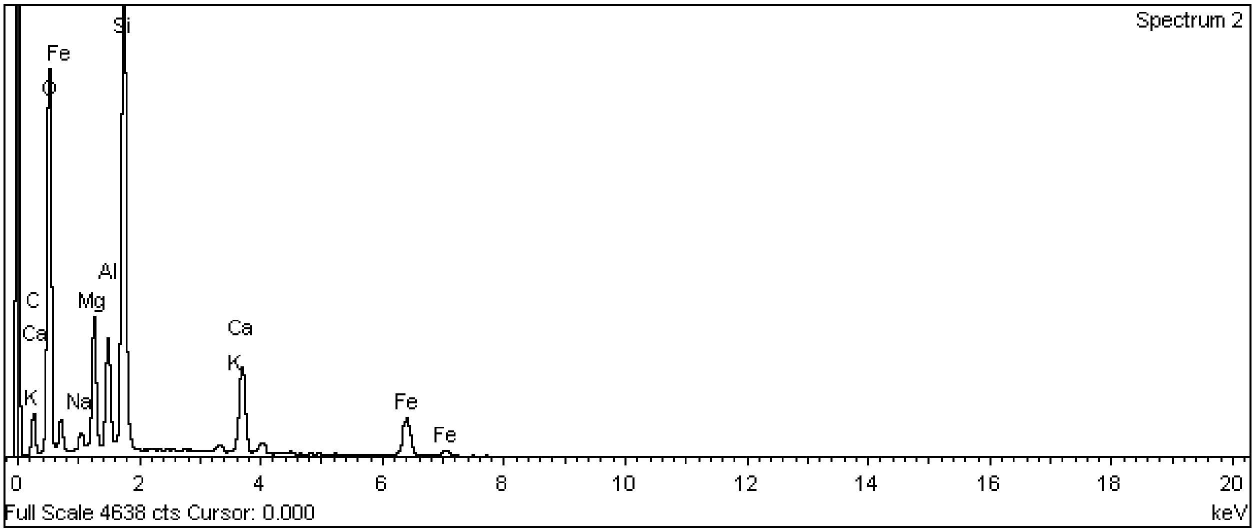

(b)

Figure S2: Spot analysis (a) and spectrum (b) of elemental composition of amphibole grain in concentrate.

Table S11: Raw data of amphibole grain generated by SEM-EDS for spectrum 2.

Element	App Conc.	Intensity Corrn.	Weight%	Weight% Sigma	Atomic%	Compd%	Formula	Number of ions
Na K	0.76	0.7734	0.98	0.09	0.99	1.32	Na2O	0.13
Mg K	5.09	0.8572	5.94	0.11	5.65	9.84	MgO	0.74
Al K	4.14	0.8850	4.68	0.10	4.01	8.84	Al2O3	0.53
Si K	19.24	0.9257	20.78	0.19	17.11	44.45	SiO2	2.26
K K	0.75	1.0364	0.73	0.07	0.43	0.88	K2O	0.06
Ca K	8.83	0.9932	8.89	0.16	5.13	12.44	CaO	0.68
Fe K	12.98	0.8989	14.44	0.35	5.98	20.64	Fe2O3	0.79
O			41.99	0.35	60.70			8.00
Total			98.42					
							Cation sum	5.18

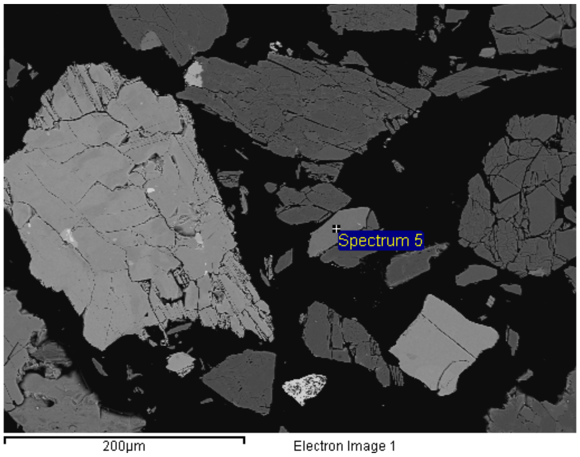

(a)

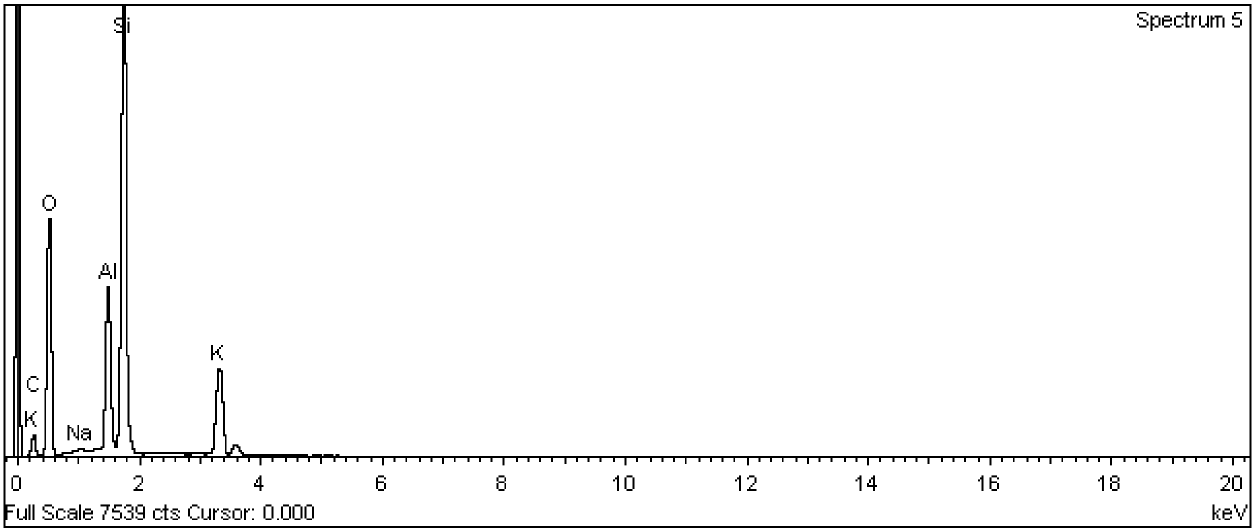

(b)

Figure S3: Spot analysis (a) and spectrum (b) of elemental composition of mica grain in concentrate.

Table S12: Raw data of mica grain generated by SEM-EDS for spectrum 5.

Element	App Conc.	Intensity Corrn.	Weight%	Weight% Sigma	Atomic%	Compd%	Formula	Number of ions
Na K	0.25	0.9318	0.27	0.07	0.24	0.37	Na2O	0.03
Al K	10.22	1.0583	9.66	0.12	7.41	18.24	Al2O3	0.96
Si K	31.64	0.9966	31.75	0.22	23.42	67.92	SiO2	3.04
K K	13.62	0.9978	13.65	0.19	7.23	16.45	K2O	0.94
O			47.65	0.28	61.69			8.00
Total			102.98					
							Cation sum	4.97

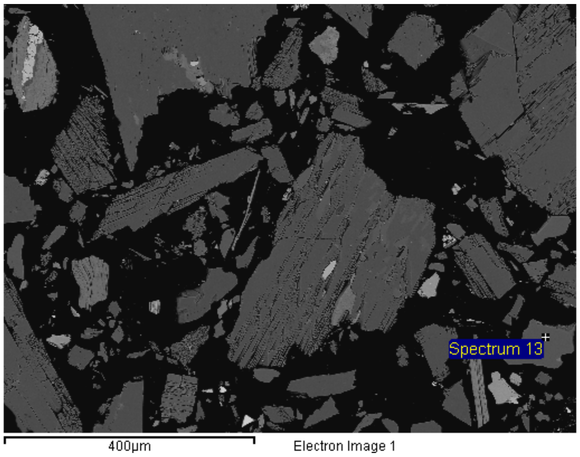

(a)

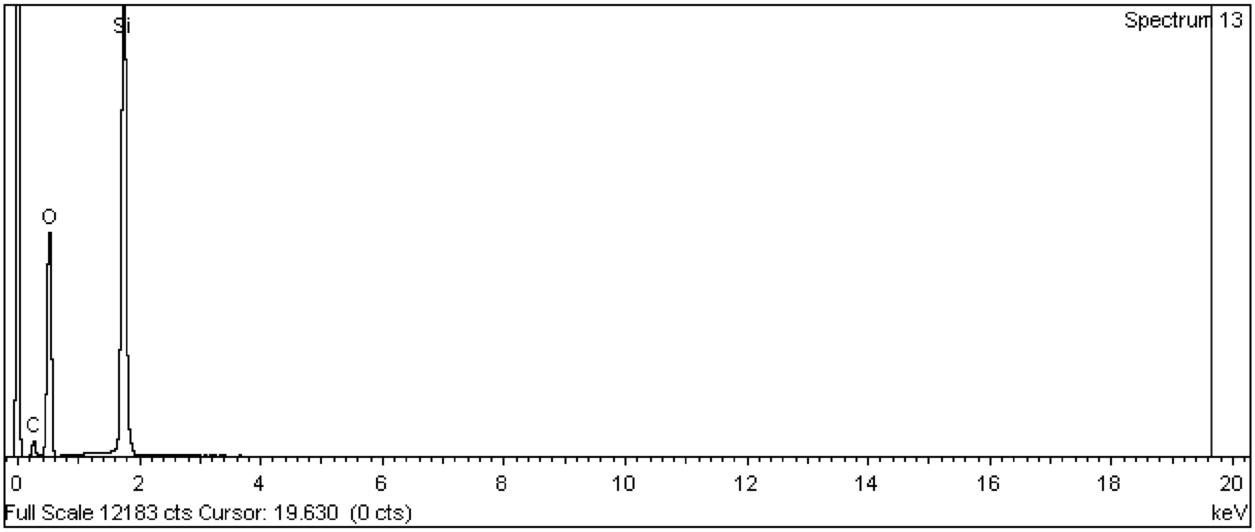

(b)

Figure S4: Spot analysis (a) and spectrum (b) of elemental composition of quartz grain in concentrate.

Table S13: Raw data of quartz grain generated by SEM-EDS for spectrum 13.

Element	App Conc.	Intensity Corrn.	Weight%	Weight% Sigma	Atomic%	Compd%	Formula	Number of ions
Si K	51.87	*1.0841*	47.84	0.25	33.33	102.34	SiO2	4.00
O			54.50	0.27	66.67			8.00
Total			102.34					
							Cation sum	4.00

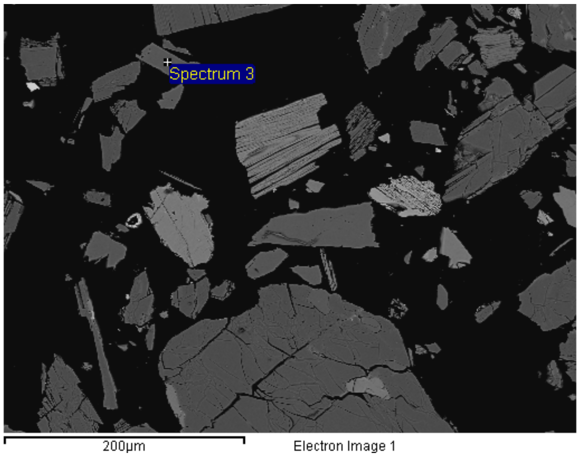

(a)

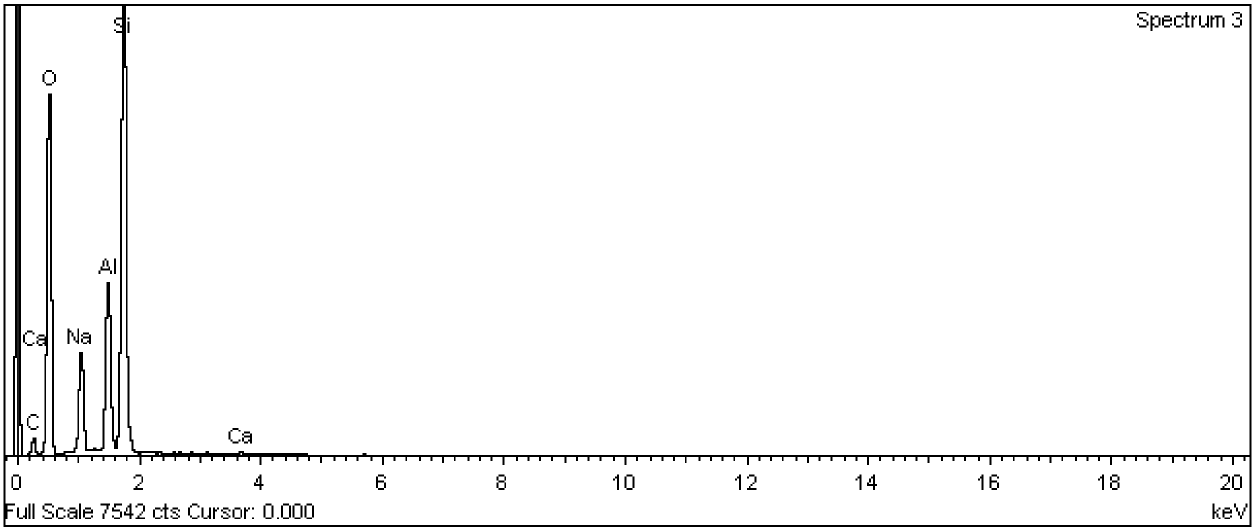

(b)

Figure S5: Spot analysis (a) and spectrum (b) of elemental composition of albite grain in concentrate.

Table S14: Raw data of albite grain generated by SEM-EDS for spectrum 3.

Element	App Conc.	Intensity Corrn.	Weight%	Weight% Sigma	Atomic%	Compd%	Formula	Number of ions
Na K	8.03	0.9852	8.16	0.14	6.97	10.99	Na2O	0.90
Al K	10.59	1.0027	10.56	0.13	7.69	19.95	Al2O3	0.99
Si K	31.65	0.9473	33.41	0.23	23.37	71.48	SiO2	3.02
Ca K	0.22	0.9489	0.23	0.06	0.11	0.33	CaO	0.01
O			50.39	0.29	61.86			8.00
Total			102.74					
							Cation sum	4.93

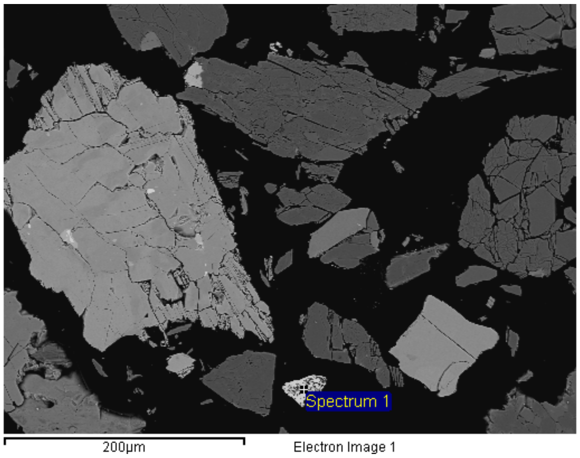

(a)

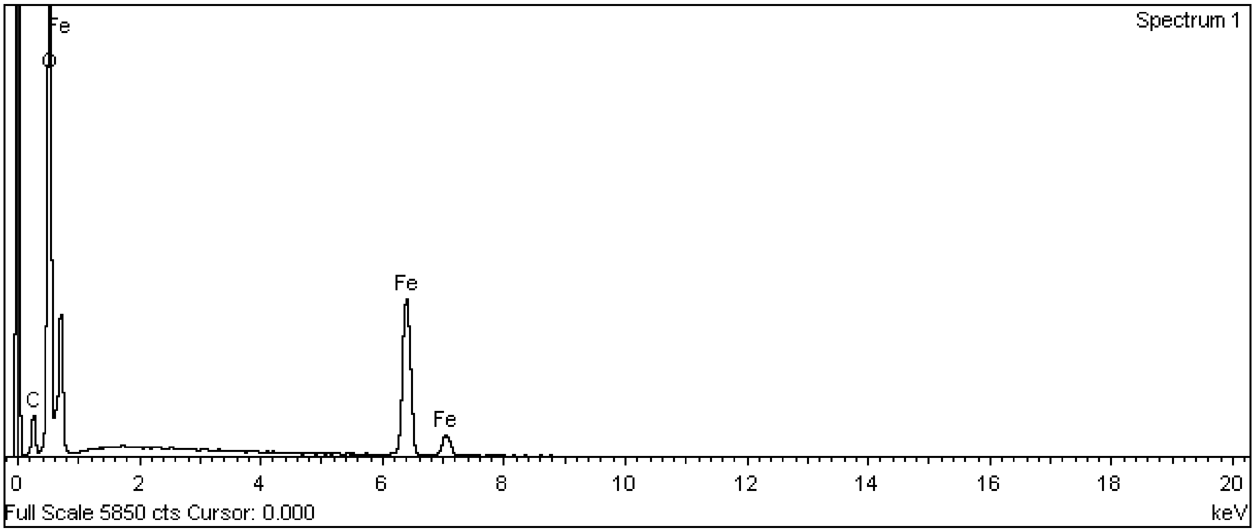

(b)

Figure S6: Spot analysis (a) and spectrum (b) of elemental composition of hematite grain in concentrate.

Table S15: Raw data of hematite grain generated by SEM-EDS for spectrum 1.

Element	App Conc.	Intensity Corrn.	Weight%	Weight% Sigma	Atomic%	Compd%	Formula	Number of ions
Fe K	70.80	0.9998	70.81	0.66	40.00	101.24	Fe2O3	5.33
O			30.43	0.43	60.00			8.00
Total			101.24					
							Cation sum	5.33

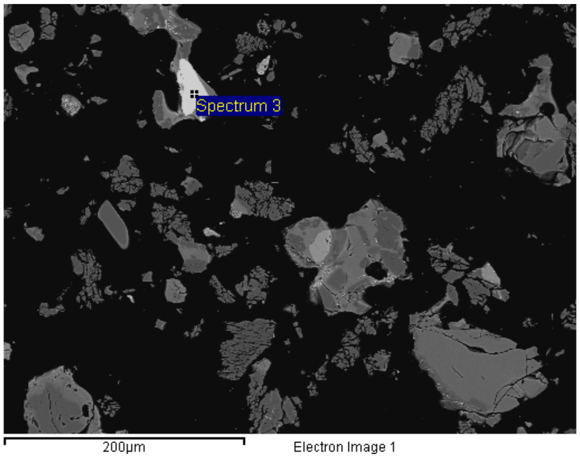

(a)

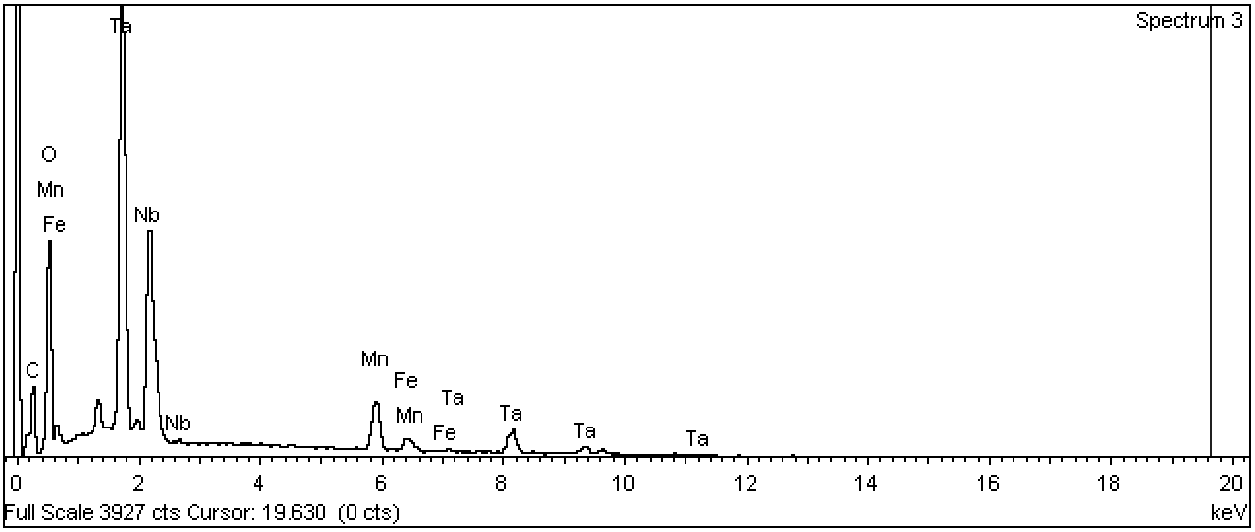

(b)

Figure S7: Spot analysis (a) and spectrum (b) of elemental composition of tantalite grain in concentrate.

Table S16: Raw data of tantalite grain generated by SEM-EDS for spectrum 3.

Element	App Conc.	Intensity Corrn.	Weight%	Weight% Sigma	Atomic%	Compd%	Formula	Number of ions
Mn K	12.43	1.1398	10.90	0.28	8.49	14.08	MnO	1.01
Fe K	2.77	1.1131	2.49	0.24	1.90	3.56	Fe2O3	0.23
Nb L	19.04	0.7267	26.21	0.54	12.06	37.49	Nb2O5	1.44
Ta M	35.55	0.8159	43.57	0.53	10.30	53.20	Ta2O5	1.23
O			25.16	0.49	67.25			8.00
Total			108.32					
							Cation sum	3.90

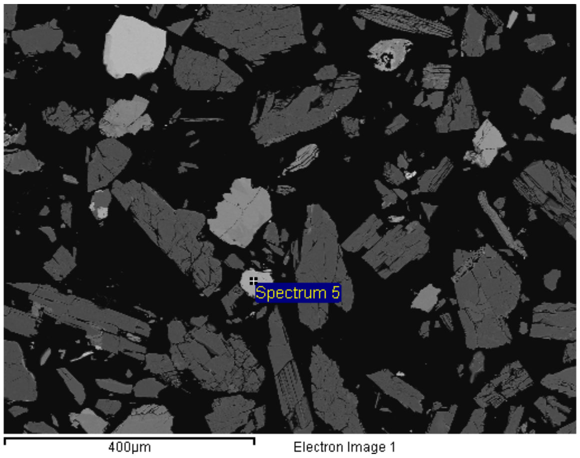

(a)

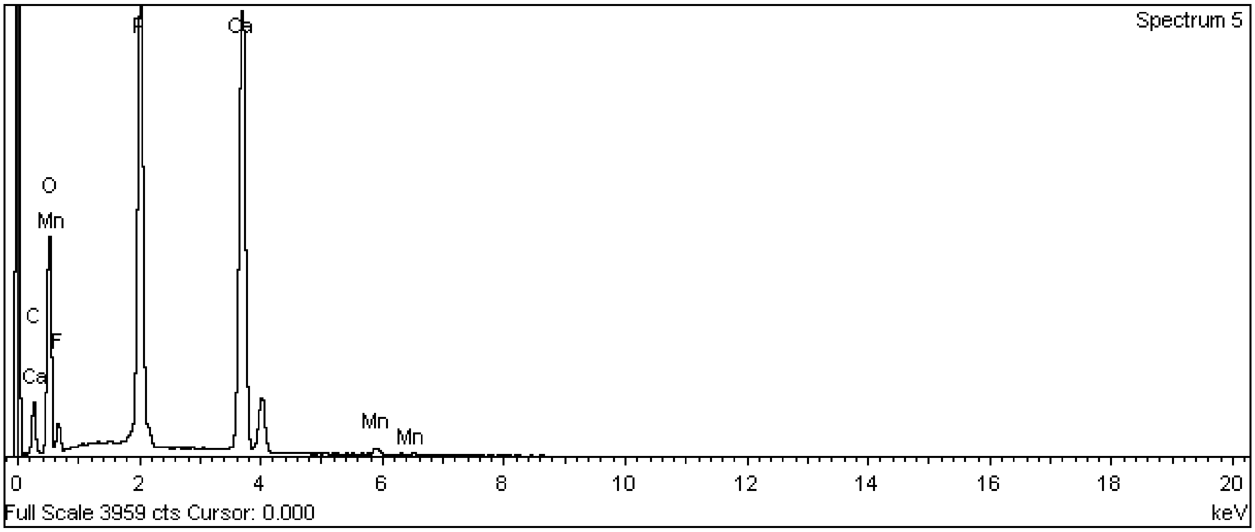

(b)

Figure S8: Spot analysis (a) and spectrum (b) of elemental composition of apatite grain in concentrate.

Table S17: Raw data of apatite grain generated by SEM-EDS for spectrum 5.

Element	App Conc.	Intensity Corrn.	Weight%	Weight% Sigma	Atomic%	Compd%	Formula	Number of ions
F K	1.52	0.2085	7.28	0.51	7.92	0.00		0.98
P K	30.90	1.4168	21.81	0.22	14.54	49.97	P2O5	1.79
Ca K	39.28	1.0094	38.91	0.29	20.05	54.44	CaO	2.47
Mn K	1.30	0.9151	1.43	0.16	0.54	1.84	MnO	0.07
O			44.11	0.32	56.95			7.02
Total			113.53					
							Cation sum	4.33

Table S18. Atomic percentage of some mineral phases identified by SEM-EDS at 900 °C.

Elements	Spot "1"	Spot "2"	Spot "3"	Spot "4"	Spot "5"	Spot "7"	Spot "9"
O	64.8	60.6	61.8	66.7	61.9	60.0	56.9
Al	11.2	0.4	7.4		7.7		
Si	23.8	19.6	23.5	33.3	23.4		
Fe	0.3	2.8				40.0	
Mg		6.8					
Ca		9.6			0.1		20.2
Mn		0.3					
Na			0.1		7.0		
K			6.5				
F							8.6
P							14.6
Sn							

Table S19. Atomic percentage of some mineral phases identified by SEM-EDS at 950 °C.

Elements	Spot "1"	Spot "2"	Spot "3"	Spot "4"	Spot "6"	Spot "7"	Spot "9"
O	64.8	60.9	61.5	66.7	2.1	60.1	56.9
Al	11.5	9.5	14.9		15.9	0.4	
Si	23.7	14.8	17.8	33.3	17.7	0.6	
Fe		4.5	0.7		0.2	38.9	
Mg						0.05	
Ca		0.3			0.2		19.8
Mn		9.9					0.6
Na			0.9		---		
K			4.3		2.3		
F							8.1
P							14.6

Table S20. Atomic percentage of some mineral phases identified by SEM-EDS at 1000 °C.

Elements	Spot "1"	Spot "2"	Spot "3,5&6	Spot "4"
O	64.6	60.7	61.8	66.7
Al	11.1	3.3	15.9	
Si	23.6	18.0	17.7	33.3
Fe	0.4	4.8	0.2	
Mg		7.1		
Ca		5.0	0.2	
Mn				
Na	0.3	0.8	2.1	
K		0.4	2.3	
Sn				

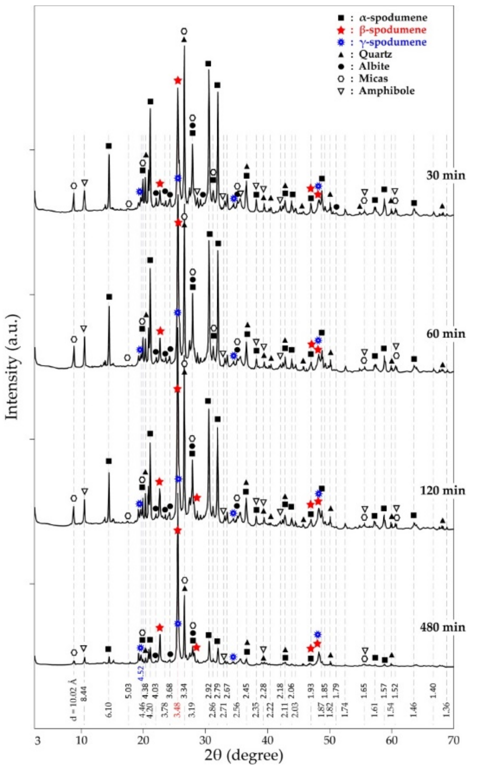
■ : α-spodumene
★ : β-spodumene
✲ : γ-spodumene
▲ : Quartz
● : Albite
○ : Micas
▽ : Amphibole
30 min
60 min
120 min
480 min
Intensity (a.u.)
2θ (degree)

(**a**)

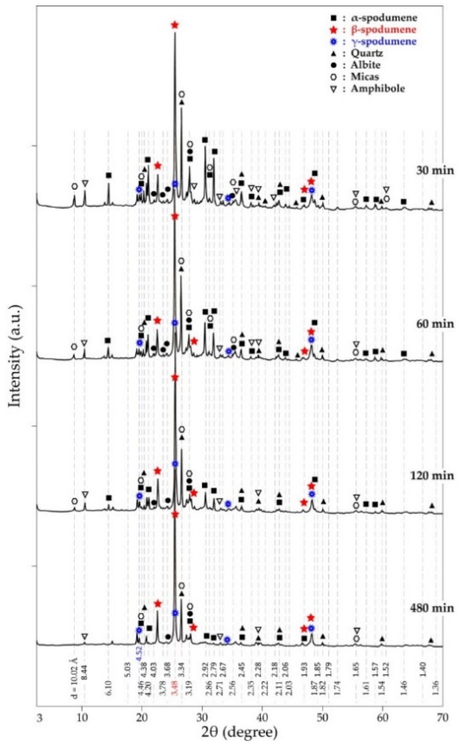
■ : α-spodumene
★ : β-spodumene
✲ : γ-spodumene
▲ : Quartz
● : Albite
○ : Micas
▽ : Amphibole
30 min
60 min
120 min
480 min
Intensity (a.u.)
2θ (degree)

(**b**)

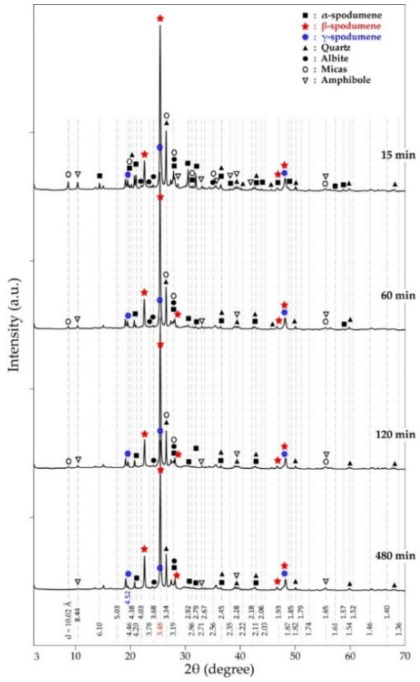
■ : α-spodumene
★ : β-spodumene
● : γ-spodumene
▲ : Quartz
● : Albite
○ : Micas
▽ : Amphibole
15 min
60 min
120 min
480 min
Intensity (a.u.)
2θ (degree)

(c)

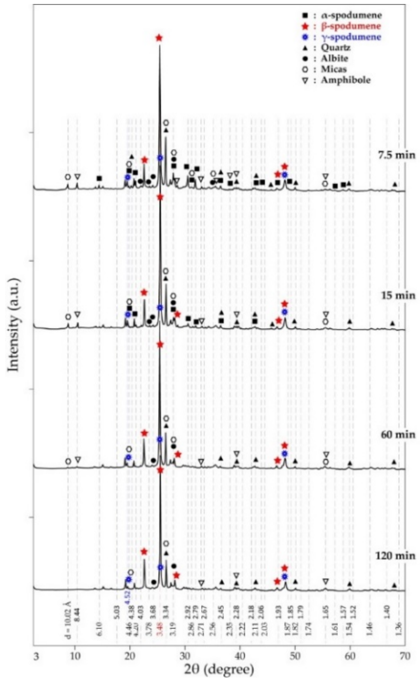
■ : α-spodumene
★ : β-spodumene
● : γ-spodumene
▲ : Quartz
● : Albite
○ : Micas
▽ : Amphibole
7.5 min
15 min
60 min
120 min
Intensity (a.u.)
2θ (degree)

(d)

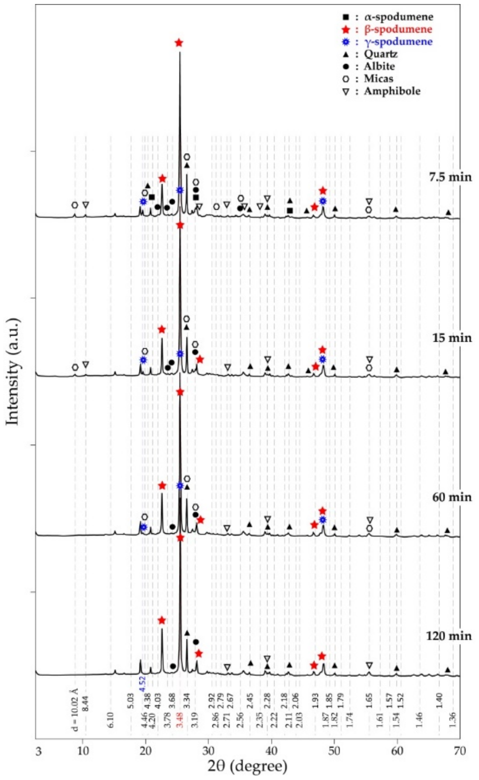

(e)

Figure S9. XRD patterns of residues obtained during treatment of concentrate in air as a function of residence time at 925 °C (a), 950 °C (b), 975 °C (c), 1000 (d), 1025 °C (e).

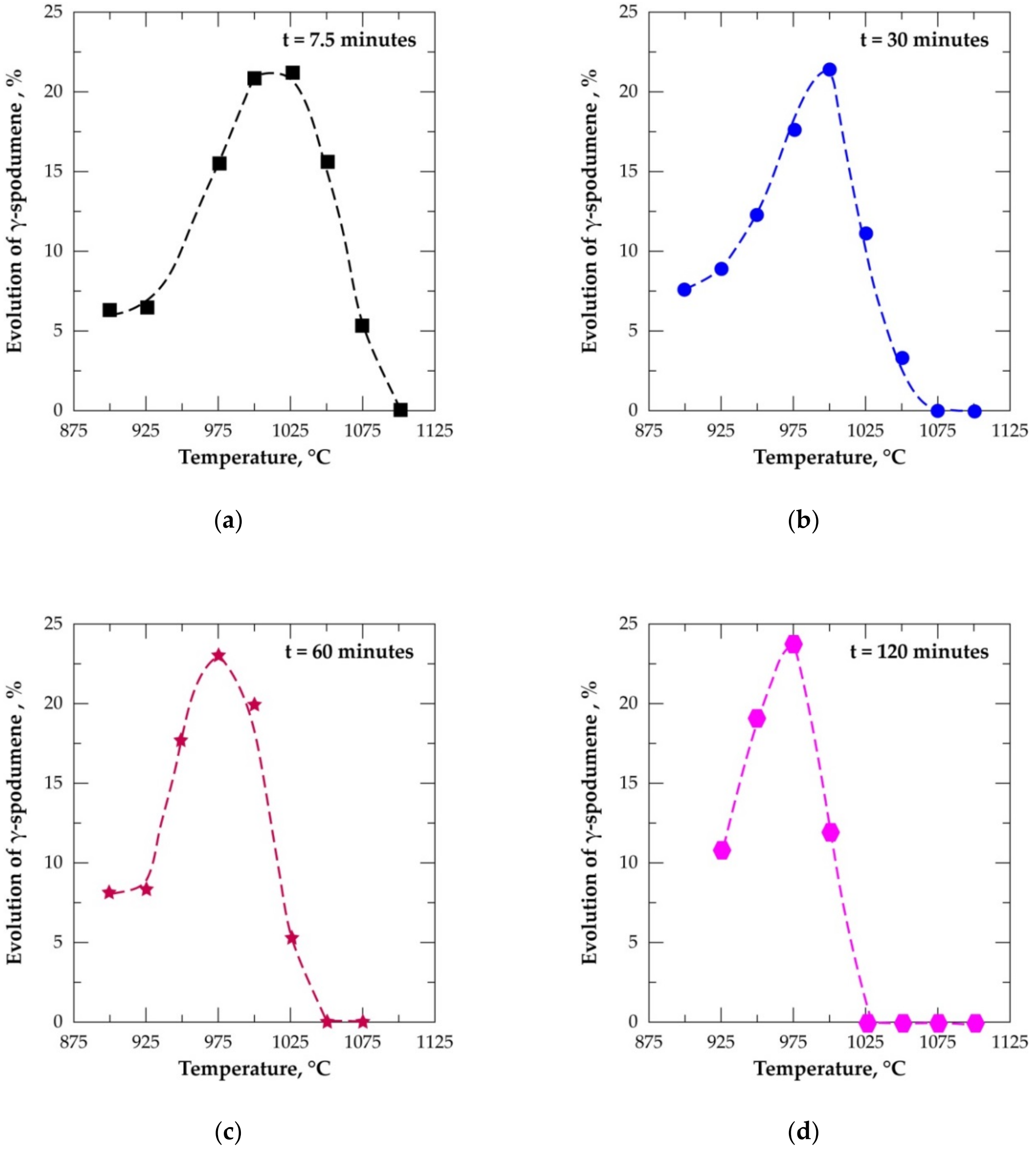

Figure S10. Evolution of the relative γ-spodumene content [$(\gamma/(\alpha+\beta+\gamma)$*100] during treatment as a function of temperature for residence times: (a) 7.5 minutes, (b) 30 minutes, (c) 60 minutes and (d) 120 minutes

Table S21. Data for determination of apparent rate constants, k1 for α-decay from Equation (3)

Time/min	**% α(t)**					**ln α(t)**				
	900°C	***925°C***	***950°C***	***975°C***	***1000°C***	***900°C***	***925°C***	***950°C***	***975°C***	***1000°C***
7.5		88.07		46.73	24.44		4.48		3.84	3.2
15		75.33		32.8	9.76		4.32		3.49	2.28
30		79.42	55.66	18.2	3.42		4.37	4.02	2.9	1.23
60	88.64	75.2	43.04	5.89	0	4.48	4.32	3.76	1.77	
120	84.77	67.06	24.47	3.71	0	4.44	4.21	3.2	1.31	
240	77.78	51.2	15.01	0	0	4.35	3.94	2.71		
480	75.09	33.92	4.51	0	0	4.32	3.52	1.51		

Table S22: Data for determination of apparent rate constants, k2 for γ-decay from Equation (4)

**Time/min**	**% γ(t)**			**ln γ(t)**		
	1000°C	1025°C	1050°C	1000 °C	1025°C	1050°C
7.5	21.01	21.17	15.63	3.04	3.05	2.75
15	20.63	7.18	7.14	3.03	1.97	1.97
30	21.58	11.31	3.42	3.07	2.43	1.23
60	19.88	5.3	0	3	1.67	
120	12.02	0	0	2.49		
240	7.47	0	0	2.01		
480	0	0	0			

Table S23: Data for calculating apparent activation energy for α-decay from Equation (5)

**Temp/°C**	**Ea for α conversion to γ and β = 652 KJmol**		
	10000/Temp (K)	k1	ln k1
900	**8.53**	0.0004	**-7.824**
925	**8.35**	0.0019	**-6.266**
950	**8.18**	0.0054	**-5.221**
975	**8.01**	0.0224	**-3.799**
1000	**7.86**	0.085	**-2.465**

Table S24: Data for calculating apparent activation energy for γ-decay from Equation (5)

Temp/°C	**Ea for γ conversion to β = 731 KJmol**		
	10000/Temp (K)	k2	ln K
1000	**7.86**	0.0048	**-5.34**
1025	**7.70**	0.0192	**-3.95**
1050	**7.56**	0.065	**-2.73**
